# Time course study of *Paenarthrobacter nicotinovorans* secondary metabolite toxicity profile

**DOI:** 10.17912/micropub.biology.000922

**Published:** 2023-09-19

**Authors:** Justyna Kakol, Mainor Vang, Drew Sausen, Tracey Steeno, Angelo Kolokithas

**Affiliations:** 1 Northeast Wisconsin Technical College, Green Bay, Wisconsin, United States

## Abstract

In previous studies *, Paenarthrobacter nicotinovorans* was isolated and screened for antimicrobial activity. Further, secondary metabolites were isolated and screened for antimicrobial activity and cytotoxicity *in vitro* . The current study determines if increased exposure of Hela cells to the secondary metabolites over time increases the cytotoxicity. The results show no detectable increase of cytotoxicity in HeLa cells.

**Figure 1. Toxicity of secondary metabolites with increasing incubation times:**
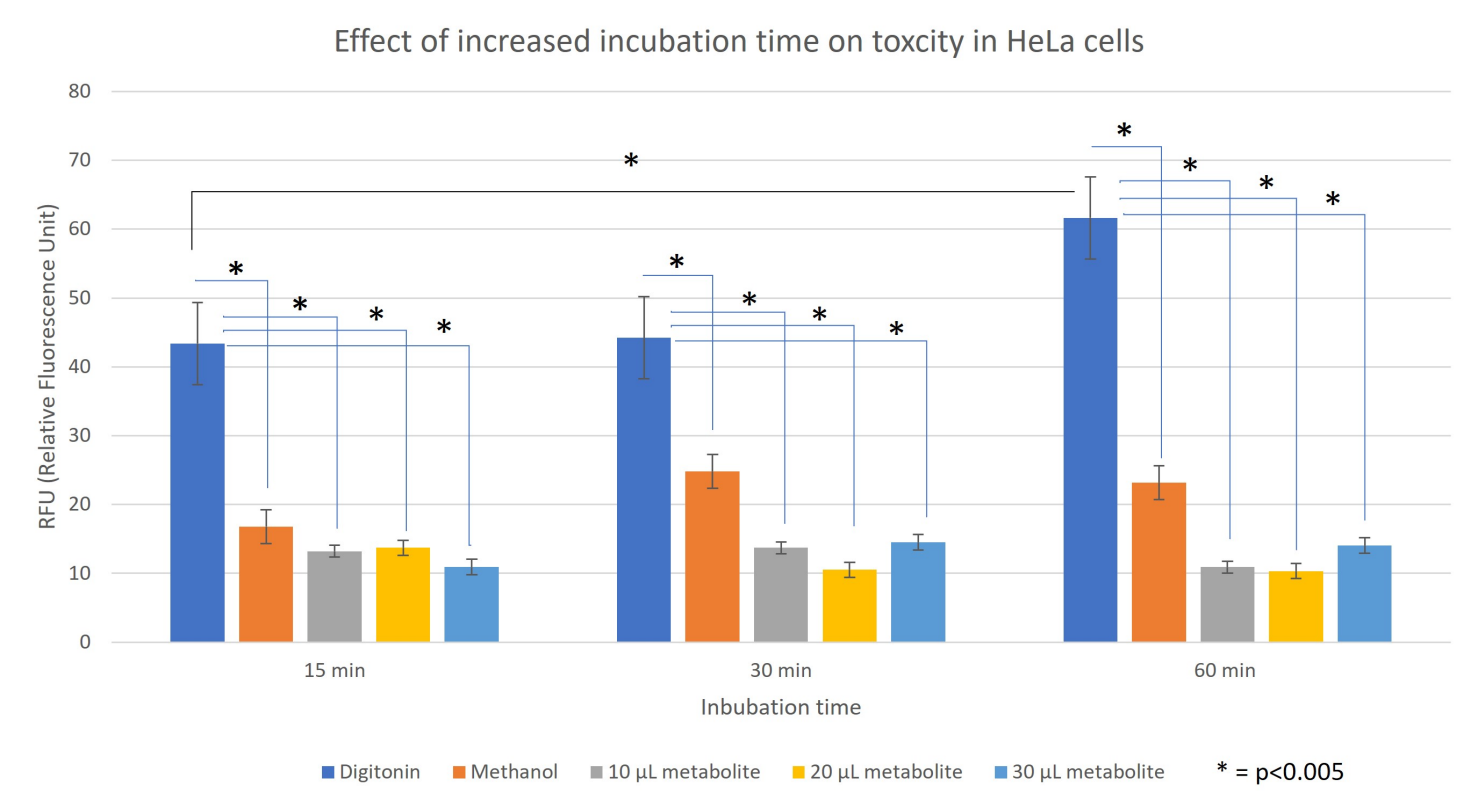
Relative fluorescence units (RFU) of HeLa cells exposed to digitonin (positive control), methanol (negative control) and three different concentrations of secondary metabolite (10 µL, 20 µL, and 30 µL) measured at three different incubation times: 15 minutes, 30 minutes, and 60 minutes. Error bars are standard error from the mean. A students t test was used to determine significance. The p values are as follows: For 15 min, Digitonin compared to methanol, p = 7.22 x 10 ^-5^ , Digitonin compared to 10μl metabolite, p = 6.68 x 10 ^-6^ , Digitonin compared to 20μl metabolite, p = 1.52 x 10 ^-5^ , Digitonin compared to 30μl metabolite, p = 1.3 x 10 ^-5^ ; for 30 min, Digitonin compared to methanol, p = 2.15 x 10 ^-4^ , Digitonin compared to 10μl metabolite, p = 4.9 x 10 ^-5^ , Digitonin compared to 20μl metabolite, p = 1.93 x 10 ^-5^ , Digitonin compared to 30μl metabolite, p = 1.4 x 10 ^-5^ ; For 60 minutes, Digitonin compared to methanol, p = 1.03 x 10 ^-5^ , Digitonin compared to 10μl metabolite, p = 6.34 x 10 ^-5^ , Digitonin compared to 20μl metabolite, p = 1.78x 10 ^-4^ , Digitonin compared to 30μl metabolite, p = 6.56 x 10 ^-5^ ; Digitonin 15 min compared to digitonin 60 min, p = 2.1x 10 ^-3^

## Description

Antibiotics are one of the most significant discoveries of the 20th century. Their discovery was a turning point in human history. Antibiotics have revolutionized medicine and saved many lives (Frieri et al, 2017) . The spread of antibiotic resistance means that antibiotics have become less effective and have lost the ability to treat serious bacterial infections (Ventola, 2015a and Ventola, 2015b). It is estimated that 1.27 million people around the world died in 2019 from infections caused by drug-resistant bacteria (Antimicrobial Resistance Collaborators, 2022) . The prediction is that antimicrobial-resistant infections will kill up to 10 million people per year by 2050 (Collier and O’Neill 2018). Many agree that there is a need for more studies on the environmental microbiomes related to discovering new antibiotics and using them to treat bacterial infections (Ventola 2015a, Ventola, 2015b, Frieri et al, 2017, Collier and O’Neill 2018, Antimicrobial Resistance Collaborators, 2022,).

Pathogens of special interest include those that make up the acronym “ESKAPE”, include six species with growing multidrug resistance and virulence such as *Enterococcus faecium, Staphylococcus aureus, Klebsiella pneumoniae, Acinetobacter baumannii, Pseudomonas aeruginosa* , and *Enterobacter species* (Rice 2008, Mulani et al, 2019, Larsson and Flach, 2022) . These ESKAPE pathogens cause the majority of nosocomial infections that are acquired in a hospital or other health care facility and are capable of “escaping” activity of many commonly used antibiotics. Moreover, they are associated with the highest risk of mortality.

The increasing problem of antibiotic resistance has been compounded by a slowdown in the discovery and development of new antibiotic strategies (Hutchings et al, 2019, Cook and Wright, 2022) . Humans are in a molecular arms race with bacteria that evolve quicker than drug development can keep up with, and new strategies are needed to address what the United Nations describes as a “fundamental threat” to humanity (Hutchings et al, 2019, Cook and Wright, 2022) . To this end, The Tiny Earth project gives the opportunity for students to be engaged in scientific research based on discovering effective antibiotics (Hurley et al, 2021) . This innovative program was launched in June of 2018 by Dr. Jo Handelsman at the University of Wisconsin-Madison. The name “Tiny Earth” reflects the tiny objects (bacteria) that are the subjects of research around the world. This global network includes thousands of students and hundreds of instructors that are involved in antibiotic discovery (Hurley et al, 2021) . Students from more than 30 countries isolate bacteria from their local soil and test them for antibiotic activity, since it is already known that over two-thirds of antibiotics come from soil bacteria or fungi (Hurley et al, 2021, Larsson and Flach, 2022) .

Northeast Wisconsin Technical College (NWTC) participates in the Tiny Earth program. In the fall semester of 2022, students from NWTC, as a part of microbiology class, had the opportunity to isolate bacteria from soil samples from various environments. Isolated bacteria were named by students’ initials and group numbers: MV2, SRC10, BB12, BS16, CM19 and CM20. Analysis of the bacterial genome sequence indicated that the MV2 bacterium isolated from the soil of a county park is a close relative of a Gram-positive bacterium called *Paenarthrobacter nicotinovorans* , which is best known for its ability to metabolize nicotine[Meng, et al, 2017, Mihasan et al, 2018, Mihasan et al, 2019, Brandsch and Mihasan, 2020, Mihasan et al, 2021, El-Sabeh, et al, 2022). This bacteria has not been studied extensively for antimicrobial properties of its secondary metabolites, however one study suggested the silver nanoparticles biosynthesized from culture supernatant of the bacteria may have some antimicrobial characteristics (Huq and Akter, 2021) .

Antimicrobials, from a medical point of view, should have a selective effect on the pathogen without being toxic for the host cells (Terreni et al, 2021) . For example, penicillin is a commonly used antibiotic that effectively kills bacteria and has low toxicity for the human body (Bentley and Bennett, 2003, Bentley 2005) . There are a wide variety of known antibiotics, but only less than 1% of antibiotics can be used in medicine (Rolain and Baquero, 2016) . Antibiotics should not have any significant harmful effects for the patient. Unfortunately, many researched antibiotics with promising antimicrobial activity were abandoned since they have negative impact on humans (Rolain and Baquero, 2016) .

Here we describe the basic toxicology of the secondary metabolites of *Paenarthrobacter nicotinovorans* with cytotoxicity testing *in vitro* .

Upon finding that the secondary metabolites were displaying antimicrobial activity (Kakol, 2023a), the toxicity of the metabolites were tested *in vitro* . The CytoTox-Fluor™ Cytotoxicity Assay was utilized to test the cytotoxicity of increasing amounts of the secondary metabolites as compared to the toxicity of digitonin, methanol, and water (Kakol, 2023b). Increasing the amount of secondary metabolites did not result in increased toxicity as compared to the digitonin control and were more like the negative controls.

To determine the effect of exposure time of the cells to the secondary metabolites, cells were exposed to either 10, 20, or 30μl of the secondary metabolites for 15, 30, or 60 minutes ( Figure 1 ). Increased exposure time did not result in the detection of increased toxicity of the secondary metabolites, whereas there was a significant increase in toxicity of the digitonin exposed samples.

At present, the world is facing an antibiotic resistance crisis. As bacterial evolution continues to outpace the development of effective antibiotic therapies, interventions such as those employed by Tiny Earth, are needed. A collaborative effort, combining technical college students across several disciplines and courses, was used to accomplish this study. Though *Paenarthrobacter nicotinovorans* is known to metabolize nicotine, its potential use for antibiotic development has only been tested by the production of silver nanoparticles, and the toxicity of these were not determined (Huq and Akter, 2021) . To this end this bacterium may represent a novel source of new antimicrobial substances, as it had activity against several pathogens (Kakol, 2023a) and did not show detectable levels of toxicity *in vitro* (Kakol, 2023b and present). Further studies are needed to elucidate the actual antimicrobial molecules that present this activity as well as further *in vivo* studies. There are several limitations to this study. Here, we provisionally identified *Paenarthrobacter nicotinovorans* using 16S rRNA sequence. A more through genetic analysis of the whole genome would help further characterize the bacterial sample. The secondary metabolites were also isolated using basic organic chemistry methods and solvents. More sophisticated isolation methods should be used in the future to isolate specific metabolites. Finally, only one measure of cytotoxicity was utilized in the present study, though there are many more available on the market to identify different types of toxicity. Further studies should utilize these measures to identify toxicity that this assay may have missed. Nonetheless, *Paenarthrobacter nicotinovorans* represents a good candidate for further antimicrobial development.

## Methods

*Cell culture*

Hela cells (ATCC #CCL-2) were maintained in Dulbecco's Modified Eagle Medium (DMEM) supplemented with 10% fetal bovine serum (FBS) and 1x Penicillin-Streptomycin-Neomycin (PSN)

*Toxicity testing*

The toxicity of the secondary metabolites from *Paenarthrobacter nicotinovorans* to the HeLa cells was measured by using a commercial CytoTox-Fluor™ Cytotoxicity Assay (Promega Catalog #G9260) according to the manufacturer’s instructions. The positive control was 300ug/ml Digitoninv (Orczyk et al, 2017) (Invitrogen, BN2006). Methanol (Sigma, 67-56-1) and DI H2O were used as vehicle and negative controls, respectively. The secondary metabolite extract of *Paenarthrobacter nicotinovorans* (10mg/ml) was used at various concentrations in the toxicity experiments, and various incubation times were used to determine if concentration or incubation time increased toxicity. Ten thousand Hela Cells per well were used in a 96-well plate and the fluorescence measurements were obtained using the Varioskan™ LUX (ThermoFischer) multimode microplate reader with the rhodamine-110 (485nmEx/520Em) filter set.

*Statistics*

Microsoft Excel was used to analyze the data. Error bars shown are standard error of the mean. Student T-tests were utilized to determine the level of significance (p values). All experiments and samples were run in triplicate.
